# Seminal fluid anaphylaxis

**DOI:** 10.1186/1710-1492-7-S2-A39

**Published:** 2011-11-14

**Authors:** Mariam Deria, Caroline Rizk, Joanne Desormeaux, Stephanie Santucci, Jacob Karsh, Jonathan Bernstein, William H Yang

**Affiliations:** 1 McMaster University, Hamilton, Ontario, Canada; 2 University of Ottawa, Ottawa, Ontario, Canada; 3 University of Cincinnati, Cincinnati, Ohio, U.S.A; 4 Allergy & Asthma Research Centre, Ottawa, Ontario, Canada, K1Y 4G2

## Background

Seminal Fluid Anaphylaxis (SFA) is a rare condition caused by IgE mediated sensitization to seminal proteins during or after coitus. It has been reported about 80 times in the medical literature ranges in symptomatology from local pruritus to serious systemic reactions. This is the first known documented case in Canada.

## Methods

One case is presented including clinical course and positive skin prick testing to husband’s seminal fluid after consultation with an allergist and obtaining written consent.

## Results

A 54 year-old woman with no atopic history was referred for evaluation after four progressive episodes of post-coital reactions with her husband. The first two episodes consisted of itching of both palms and soles of feet with minimal vaginal pruritus. After abstaining, a third episode a month later consisted of hives on the patient’s legs and back with significant vaginal pruritus. The fourth episode the patient developed vaginal and generalized pruritus, urticaria, palpitations and trouble breathing through her nose, immediately post-coital. All symptoms resolved spontaneously with no treatment. Skin prick testing was conducted using the partner’s seminal fluid to confirm the clinical suspicion of SFA, producing a wheal of 8mm and flare of 5cm.

## Conclusions

The partner’s seminal fluid will be fractionated to determine the specific amino acid sequence of interest by serum-specific IgG/IgE by ELISA to the seminal plasma and plasma proteins. An SDS-PAGE and IgE immunoblot assay will confirm specific activity to the semen. Of clinical relevance, mass production of this protein will be used for local or systemic immunotherapy to prevent future SFA.

